# Cryo-EM reveals conformational flexibility in apo DNA polymerase ζ

**DOI:** 10.1016/j.jbc.2021.100912

**Published:** 2021-06-24

**Authors:** Chloe Du Truong, Theodore A. Craig, Gaofeng Cui, Maria Victoria Botuyan, Rachel A. Serkasevich, Ka-Yi Chan, Georges Mer, Po-Lin Chiu, Rajiv Kumar

**Affiliations:** 1 School of Molecular Sciences, The Biodesign Institute, Arizona State University, Tempe, Arizona, USA; 2 Nephrology and Hypertension Research, Division of Hypertension and Nephrology, Department of Medicine, Mayo Clinic, Rochester, Minnesota, USA; 3 Department of Biochemistry and Molecular Biology, Mayo Clinic, Rochester, Minnesota, USA; 4 Department of Cancer Biology, Mayo Clinic, Rochester, Minnesota, USA; 5 Biodesign Center for Structural Applied Discovery, Arizona State University, Tempe, Arizona, USA

**Keywords:** DNA repair, mutagenesis, Polζ, Rev7, Rev1, single-particle cryo-EM, translesion synthesis, shieldin 3, SHLD3, shieldin complex, 2D, two-dimensional, 3D, three-dimensional, BLI, biolayer interferometry, cryo-EM, cryogenic electron microscopy, dCMP, deoxycytidine monophosphate, dCTP, deoxycytidine triphosphate, FSC, Fourier-shell correlation, PCNA, proliferating cell nuclear antigen, RBM, Rev7-binding motif, SHLD3, shieldin-3, TLS, translesion synthesis

## Abstract

The translesion synthesis (TLS) DNA polymerases Rev1 and Polζ function together in DNA lesion bypass during DNA replication, acting as nucleotide inserter and extender polymerases, respectively. While the structural characterization of the *Saccharomyces cerevisiae* Polζ in its DNA-bound state has illuminated how this enzyme synthesizes DNA, a mechanistic understanding of TLS also requires probing conformational changes associated with DNA- and Rev1 binding. Here, we used single-particle cryo-electron microscopy to determine the structure of the apo Polζ holoenzyme. We show that compared with its DNA-bound state, apo Polζ displays enhanced flexibility that correlates with concerted motions associated with expansion of the Polζ DNA-binding channel upon DNA binding. We also identified a lysine residue that obstructs the DNA-binding channel in apo Polζ, suggesting a gating mechanism. The Polζ subunit Rev7 is a hub protein that directly binds Rev1 and is a component of several other protein complexes such as the shieldin DNA double-strand break repair complex. We analyzed the molecular interactions of budding yeast Rev7 in the context of Polζ and those of human Rev7 in the context of shieldin using a crystal structure of Rev7 bound to a fragment of the shieldin-3 protein. Overall, our study provides new insights into Polζ mechanism of action and the manner in which Rev7 recognizes partner proteins.

Environmental factors such as chemicals and ultraviolet light as well as metabolic processes cause cellular DNA damage and genomic instability, resulting in DNA lesions that can stall replicative DNA polymerases Polδ or Polε (1, 2, 3, 4). Eukaryotes and prokaryotes have evolved a mechanism, called translesion synthesis (TLS), which allows the replication machinery to bypass DNA lesions. This process can be mutagenic due to the misincorporation of nucleotides across the lesion site (4, 5, 6, 7, 8, 9).

There are three known TLS polymerases in *E. coli* and 15 in eukaryotes (10). One of the eukaryotic TLS polymerases, Rev1, serves as a scaffolding protein that recruits other TLS polymerases to replication forks (4, 5, 11, 12, 13, 14, 15, 16). Rev1 interacts with other TLS polymerases *via* distinct interfaces in its C-terminal domain (5, 12, 17). Rev1 also possesses deoxycytidine monophosphate (dCMP) transferase activity and functions as an insertion TLS polymerase, which incorporates nucleotides (usually deoxycytidine triphosphate or dCTP) opposite damaged and nondamaged guanines (4, 5, 11, 12, 18, 19).

Among Rev1 partner TLS polymerases, Polζ has been extensively studied both in human and in yeast and shown to mediate damage-induced mutagenesis (20, 21, 22, 23). It belongs to the B family of polymerases and is composed of subunits Rev3, Rev7, Pol31, and Pol32 (24, 25) (Fig. 1*A*). Polζ has lower processivity but higher fidelity than the Y family of polymerases (24, 25, 26, 27). Rev3 is the catalytic subunit of Polζ and can perform its function alone (26). The accessory subunit Rev7 increases Rev3 activity by at least 20-fold, suggesting an enhancing role in Polζ processivity (26). Two three-dimensional (3D) structures of *Saccharomyces cerevisiae* Polζ were recently determined in the presence of DNA oligomers of different lengths using single-particle cryogenic electron microscopy (cryo-EM) (28). Although the cryo-EM density for DNA was not detectable in one of the DNA-Polζ complex structures, the DNA might still influence the structure. Without a true apo Polζ structure, our mechanistic understanding of TLS initiation by Polζ remains incomplete.

**Figure 1:**
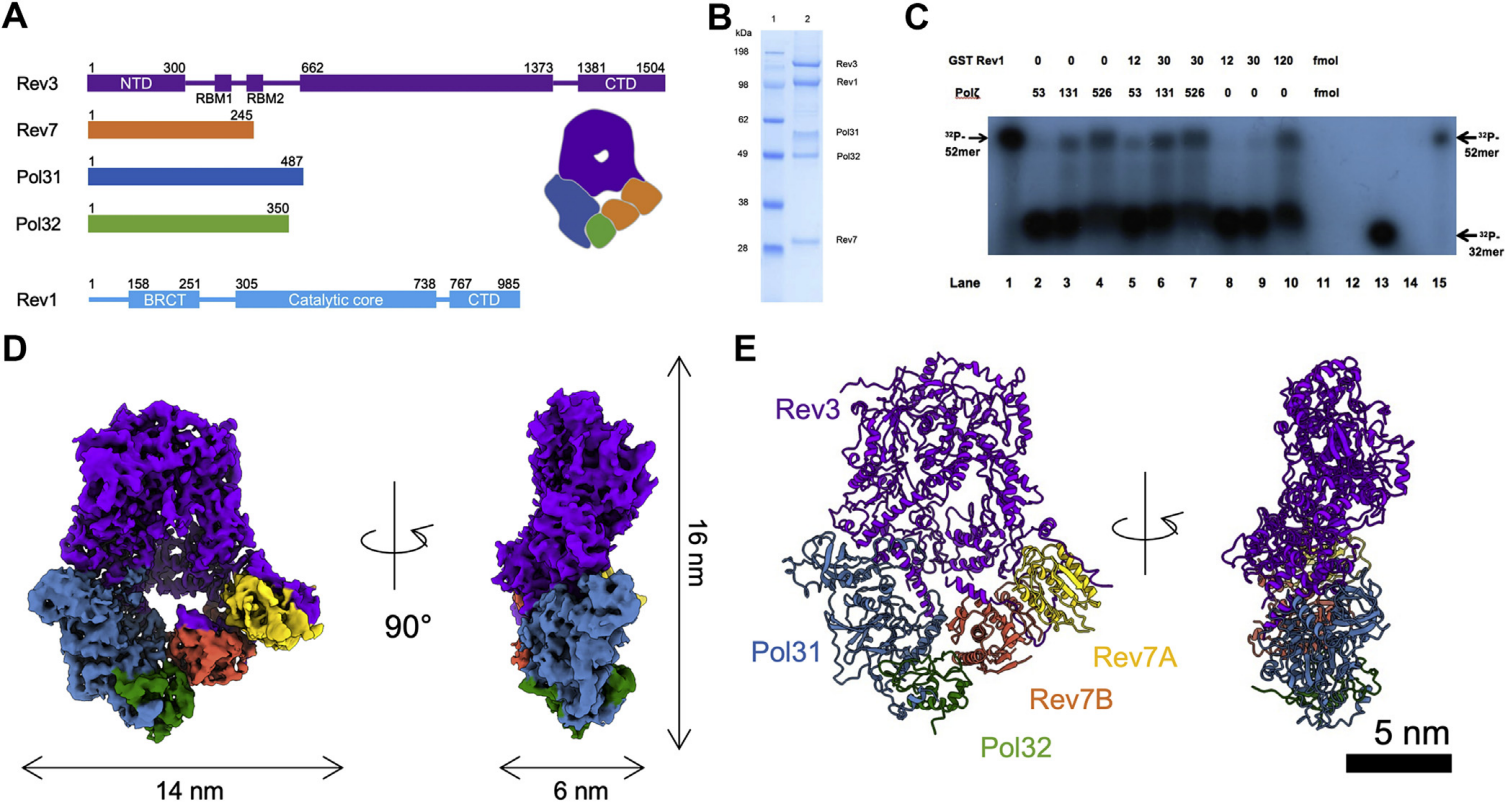
**Analysis of the interaction between yeast Polζ and Rev1 TLS polymerases and cryo-EM reconstruction of apo Polζ.***A*, schematic of the structural organization of yeast Polζ subunits and Rev1. Rev3, Rev7, Pol31, Pol32, and Rev1 are shown in *purple*, *orange*, *steel blue*, and *green*, respectively. NTD and CTD stand for N-terminal and C-terminal domains, respectively. RBM1 (517–540 aa) and RBM2 (599–623 aa) are Rev7-binding motifs 1 and 2. BRCT stands for BRCA1 C-terminal domain. *B*, SDS-PAGE analysis of the coexpression of the Polζ and Rev1 TLS polymerases in yeast. The gel was stained with Coomassie blue. *C*, Polζ, Rev1, or a mixture of the two polymerases was used to extend a ^32^P oligonucleotide 32-mer primer annealed to a 52-mer template strand with an abasic site. Low amounts of either polymerase (lanes 2 and 8) did not extend the primer but Polζ combined with Rev1 allowed extension past the abasic site (lanes 5 and 6). *D*, cryo-EM density of the apo Polζ enzyme complex. *E*, atomic model of the apo Polζ enzyme complex. The coordinates of Rev3 (*purple*), Rev7 (*gold* (Rev7A), and *orange* (Rev7B)), Pol31 (*steel blue*) and Pol32 (*green*) were built along the determined cryo-EM densities. Scale bar indicates 5 nm.

In this study, we expressed and purified Polζ from *S. cerevisiae* and characterized its association with Rev1. We verified that Rev1 binds Polζ with high affinity and functions synergistically with Polζ to extend DNA beyond an abasic lesion. We then used single-particle cryo-EM to visualize the structure of the pentameric apo Polζ holoenzyme comprising two Rev7 subunits and one subunit each of Rev3, Pol31, and Pol32 arranged around a central canal. By comparison with the previously determined cryo-EM structures of DNA-bound Polζ, we assessed possible conformational changes in Polζ associated with DNA binding to gain mechanistic insights into the initiation phase of Polζ-mediated TLS.

## Results

### Characterization of the Polζ complex and its interaction with Rev1

We purified the Polζ complex from *S. cerevisiae* using GST-affinity and metal-chelation chromatography. For a typical purification, we processed 0.5 to 2.5 kg of yeast cells, yielding approximately 250 μg of purified Polζ per kg of cells. SDS-PAGE of the purified protein showed four bands at 175, 55, 49, and 28 kDa (Fig. S1*A*). Mass spectrometric analysis indicated that the 175 kDa band was the Rev3 catalytic subunit; the 55 kDa band was the Pol31 subunit; the 49 kDa band was the Pol32 subunit; and the 28 kDa band was the Rev7 processivity subunit (Fig. S1, *B* and *C*). The quality of the protein complex was assessed using negative-stain electron microscopy (EM) (Fig. S1, *D* and *E*). The EM images showed a stable and homogeneous protein complex, and the two-dimensional (2D) class averages showed clear features of Polζ in different views. Thus, the apo Polζ protein complex can be stably formed in the absence of DNA oligomers.

Rev1 was purified using GST-affinity chromatography. SDS-PAGE showed a major band at 139 kDa and a minor band at 100 kDa (Fig. S2, *A*–*C*). Analysis of both bands by mass spectrometry produced a sequence compatible with full-length Rev1 with 90 to 95% coverage, suggesting that the difference in gel mobility was due to posttranslational modifications (Fig. S2, *A*–*C*).

We tested the binding of Rev1 to Polζ using nickel-affinity chromatography and showed that the two proteins copurified and formed a tight complex (Fig. 1*B*). The Pol32 subunit of Polζ has a heptahistidine-tag. Using biolayer interferometry (BLI), we measured a dissociation constant (*K*_D_) of 0.11 ± 0.12 μM (mean ± sd of *n* = 4 independent experiments) for the Rev1-Polζ complex.

Next, we tested the capacity of Polζ and Rev1 to extend DNA beyond an abasic lesion. In the absence of Rev1, low concentration of Polζ (2.65 nM) could not extend DNA but when a small amount of Rev1 (0.6 nM, final concentration) was added, DNA extension was detected (Fig. 1*C*). Rev1 by itself at low concentrations (0.6 and 1.5 nM) could not extend DNA (Fig. 1*C*). Therefore, Polζ and Rev1 polymerases have a synergistic effect on DNA extension. However, at higher concentrations, Rev1 (6 nM) and Polζ (6.6 and 26.3 nM) are each capable of extending DNA without the other polymerase. At elevated concentrations, the two enzymes might have redundant functions or synergize with copurified endogenous yeast enzymes.

### Visualization of the apo Polζ using single-particle cryo-EM

We used single-particle cryo-EM to study the structural organization of bioactive Polζ that included all five subunits (Fig. 1*A*). We obtained a consensus 3D cryo-EM reconstruction at 4.11 Å resolution, determined by the golden standard Fourier-shell correlation (FSC) method at a cutoff of 0.143 (29) (Fig. S3, *A* and *B*). Local resolution analysis of the 3D density showed an anisotropic resolution distribution, implying flexibility for the apo Polζ enzyme (Fig. S3*C*). To improve the quality of the local densities, we performed signal subtraction and focused refinement procedures on the Rev3 and on Rev7-Pol31-Pol32 subunits, separately (30). We then generated cryo-EM densities of the two separate systems at higher resolutions (3.65 Å for the Rev3 density and 3.72 Å for the Rev7-Pol31-Pol32 density) (Fig. S4). The atomic coordinates for Rev3, Rev7, Pol31, and Pol32 we modeled into the cryo-EM density maps (see Experimental procedures) have well-defined rotameric side chain conformations (Fig. 1*D* and Fig. S5). We can also identify the cryo-EM density of the [4Fe-4S] cluster in Rev3, which is essential to the catalytic activity of Rev3 (31). The cryo-EM structure determination statistics are summarized in Table 1.

**Table 1:** Statistics of the single-particle cryo-EM structure determination of the apo DNA polymerase ζ complex of *Saccharomyces cerevisiae*

Protein	Apo DNA polymerase ζ (Polζ) (PDB: 7LXD; EMDB: EMD-23570)
Data collection	
Electron microscope	Thermo Fisher/FEI Titan Krios TEM
Accelerating voltage (kV)	300
Spherical aberration constant (mm)	2.7
Detector camera	Gatan K2 Summit DED camera
Defocus (μm)	−0.6 to −3.0
Nominal magnification	48,077×
Physical pixel size (Å/pixel)	1.025
Image dose (e^−^/Å^2^)	44.3
Image processing	
Number of movies	11,698
Number of particles selected (initial)	1,658,585
Number of particles used for final 3D density (final)	213,120
Spatial frequency at FSC of 0.143 (Å^−1^)	4.11
Imposed symmetry	C1
Sharpening *b*-factor (Å^2^)	−162.8
Modeling	
Initial model used (PDB code)	6V8P
Model composition	
Nonhydrogen atoms	17,456
Protein residues	2093
Ligands	8
*B* factors (Å^2^)	
Protein	120.3
Ligands	94.0
RMS deviations	
Bond length (Å)	0.006
Bond angle (°)	1.299
Clash score	9.33
MolProbity score	1.62
Rotamer outlier (%)	0.00
Ramachandran plot (%)	
Disallowed	0.00
Allowed	2.68
Favored	97.32

The five subunits of apo Polζ organize into a ring-like structure (Fig. 1*D*). This arrangement of subunits is the same as that in previous Polζ structures determined in complex with DNA oligomers of different lengths (PDB codes: 6V8P and 6V93) (28). The subunits Pol31 and Pol32 bind the C-terminal domain of Rev3. In particular, the interaction of Pol31 is stabilized by the iron–sulfur cluster (4Fe-4S) in Rev3 (23). Different from the aforementioned Polζ cryo-EM structures (28), our structure is purely the apo form of Polζ (Fig. S6*A*). Superposition of the apo and DNA-bound Polζ structures shows a concerted rigid-body movement of several Polζ regions associated with DNA binding (Fig. S6*A*).

The Pol31 and Pol32 subunits are also essential to Polδ polymerase, and the spatial arrangement of these subunits in apo Polζ is the same as that in Polδ (32, 33). The difference between Polδ and Polζ is the addition of two Rev7 subunits in Polζ interfacing with Rev3, Pol31, and Pol32 (Fig. 1*D*). Rev3 interacts directly with Pol31 and Rev7 but does not contact Pol32 (Fig. 1*D*).

### Conformational changes in Polζ upon DNA binding

**Figure 2:**
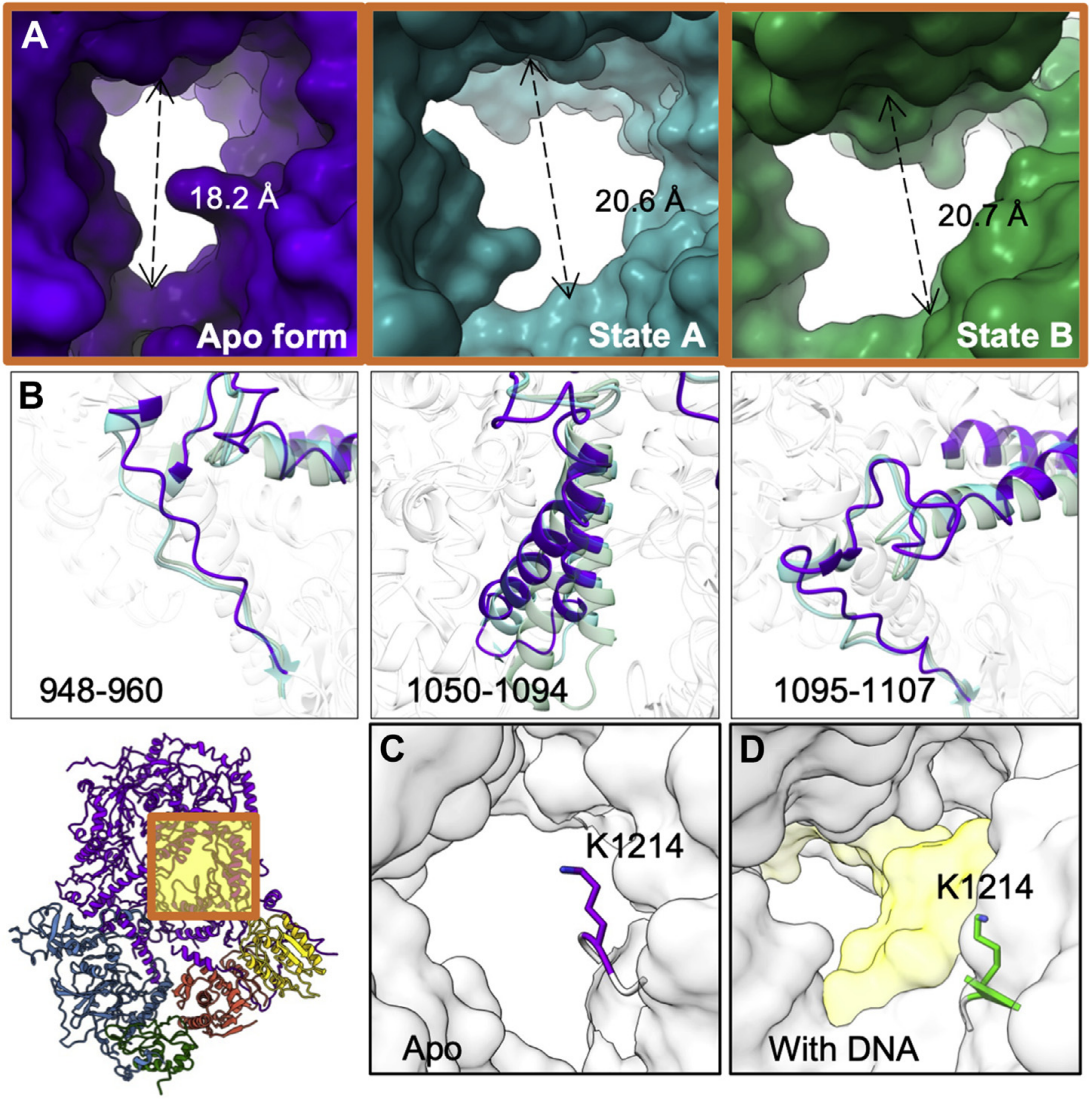
**Central channel of the Polζ enzyme complex and conformational changes associated with DNA binding.***A*, central channel of Rev3 for DNA binding. Surfaces of the apo form and states A and B are shown in *purple*, *cyan* and *green*, respectively. The sizes of the channel openings were measured as 18.2 Å, 20.6 Å, and 20.7 Å for the apo form and states A and B, respectively. *B*, conformational changes of Rev3 loops (948–960 aa, 1050–1094 aa and 1095–1107 aa) upon DNA oligomer binding. The indicated loops of the apo form and DNA-bound Polζ are highlighted in *purple* and *sea green*, respectively. *C*, rotameric conformation of Rev3 K1214. The side chain of K1214 primary amine points toward the center of the hole in the apo form, whereas it moves away when the Polζ complex binds DNA. *D*, side chain of K1214 in DNA-bound Polζ. DNA backbone is colored *yellow*. The positively charged K1214 side chain points away from the negatively charged sugar-phosphate backbone.

The ring structure of Polζ harbors a narrow central channel for oligonucleotide binding (Fig. 1, *D* and *E* and 2). Upon DNA binding, concerted movements of three Rev3 loops (948–960 aa, 1050–1094 aa, and 1095–1107 aa) increase the size of the DNA-binding channel opening, from a closed to an open state (Fig. 2, *A* and *B*). The loop (1050–1094 aa) near the central channel experiences an up-to-down movement upon DNA binding (Fig. 2*B* and Fig. S6*C*) while adjacent flexible loop region (1326–1344 aa) folds into a short α-helix upon DNA binding (Fig. S6*D*). Such changes could be a result of DNA insertion and Polζ processivity (Fig. 2*B* and Fig. S6, *C* and *D*). The channel opening sizes of apo Polζ and Polζ states A and B are 18.2 Å, 20.6 Å, and 20.7 Å, respectively (Fig. 2*A*). State A represents the short DNA oligomer-bound Polζ (PDB: 6V8P), and state B represents the longer DNA oligomer-bound Polζ (PDB: 6V93). Thus, the channel of Polζ is closed in the absence of DNA. It is possible that the presence of a short DNA oligomer induces structural changes in the Rev3 loops at residues 948 to 960 and 1095 to 1107 (Fig. 2*B*) and allows the DNA oligomer to initiate a contact with the Rev3 active site, opening up the central channel for DNA processing in translesion synthesis. The channel size change seems to be solely linked to local structural variations of the Rev3 loops, independent of other Polζ subunits. A previous study showed that Rev3 alone has catalytic activity without binding any other subunits (26). The initiation phase of DNA translesion synthesis may therefore only require Rev3 and DNA. It is possible that other subunits in the complex, that is, Rev7, Pol31, and Pol32, play a role in regulating the Rev3 activity or in interacting with other proteins. To further illustrate the movements associated with the DNA-bound and unbound states of Polζ, we morphed the three cryo-EM structures of apo Polζ and DNA-bound Polζ states A and B together and generated a movie that highlights concerted movements of the local regions (Movie S1).

Our apo structure shows that one lysine residue (K1214) of Rev3 protrudes up and points toward the pore where nucleotide insertion occurs (Fig. 2*C*). This lysine is conserved in TLS polymerases across different species (Fig. S7). When a DNA oligomer is present, K1214 moves away from the pore and leaves space for DNA binding. The positively charged K1214 (PDB: 6V93; state B) is close to but does not interact with the negatively charged DNA polar sugar-phosphate backbone in the minor groove (Fig. 2*D*) (28). This may suggest less friction for DNA binding and Polζ processivity due to removal of electrostatic contacts between K1214 and DNA. Because K1214 does not strongly interact with neighbor residues, it is possible that different conformations of K1214 coexist and that single-particle cryo-EM only captured one of the rotameric states. Thus, K1214 may play a role in gating the oligonucleotide passage.

### Dimerization of Rev7 subunits in the apo Polζ complex

**Figure 3:**
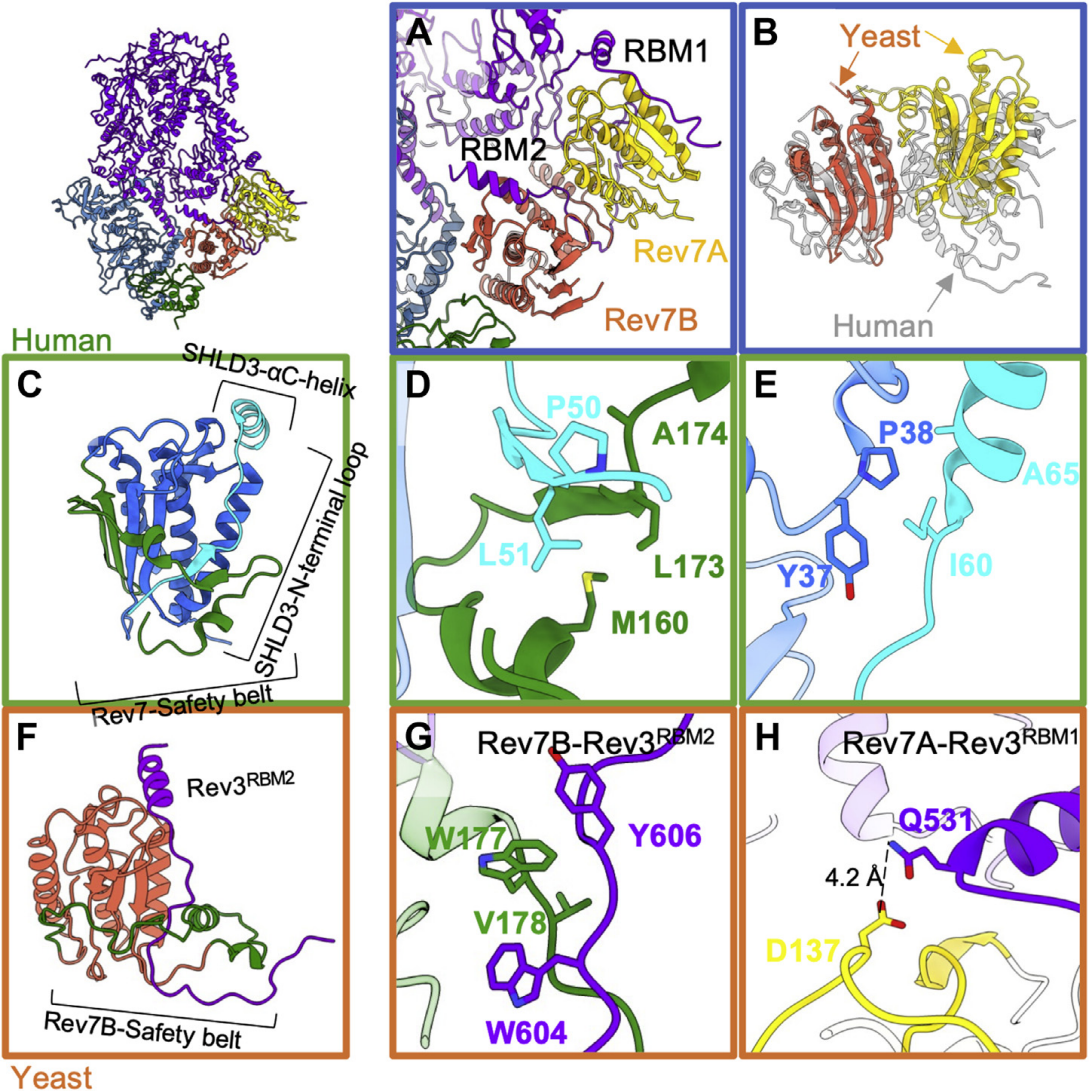
**Comparison of yeast and human Rev7 dimerization states and binding modes.***A*, Rev7 dimer in the yeast apo Polζ complex. Rev7A (*yellow*) and Rev7B (*orange*) bind to Rev7-binding motif 1 (RBM1) and 2 (RBM2) of Rev3 (*purple*), respectively. The RBM2 motif is sandwiched between the two Rev7 protomers, limiting its structural mobility. The RBM1 motif is more accessible than RBM2 suggesting less spatial restriction. *B*, overlay of yeast and human Rev7 dimers with yeast Rev7B (*orange*) and one of the human Rev7 protomers (*gray*) oriented in the same manner. This comparison highlights the radically different orientations of the two protomers in yeast (*orange* and *yellow*) and human (*gray*) Rev7. *C*, crystal structure of human Rev7(R124A) in complex with SHLD3 (41–74 aa). Rev7 core, safety belt region, and SHLD3 are shown in *blue*, *green* and *cyan*, respectively. *D*, safety belt region of human Rev7 (M160, L173 and A174) interacts with the N-terminal loop of SHLD3 (P50 and L51). *E*, interaction between human Rev7 (Y37 and P38) and SHLD3 (I60 and A65). *F*, structure of yeast Rev7B-Rev3^RBM2^ within the apo Polζ complex. Rev7B, safety belt region, and Rev3^RBM2^ are shown in *orange*, *green* and *purple*, respectively. *G*, interaction between the safety belt of yeast Rev7B (W177 and V178) and Rev3^RBM2^ motif (W604 and Y606). *H*, interaction between yeast Rev7A (D137) and Rev3^RBM1^ (Q531). Rev7A is colored *yellow*.

Polζ has two identical Rev7 accessory subunits, which are important for promoting Polζ catalytic activity and interacting with other proteins that regulate the activity of Polζ (26, 34, 35, 36, 37, 38). The Rev7 homodimer is essential for the stability and function of the Polζ complex (39, 40). Rev7 directly binds Rev1, and this interaction is important for TLS regulation (39, 41, 42). In yeast, Rev7 also stimulates the catalytic activity of Rev3 (26). Within the Polζ complex, the two subunits of Rev7, Rev7A, and Rev7B bind the Rev7-binding motifs 1 (RBM1; 517–540 aa) and 2 (RBM2; 599–623 aa) of Rev3, respectively (Fig. 3*A*). Additionally, Rev7B bridges Pol31 to Pol32 (6) (Fig. 3*A*). These interactions bring together the accessory subunits Rev7, Pol31, and Pol32 and the catalytic subunit Rev3.

Rev7 is a member of the HORMA (Hop1, Rev7, and Mad2 proteins) domain protein family (43). HORMA proteins share a common core structure composed of three β-strands flanked by three α-helices and a “safety-belt” region, which closes around target proteins. The safety-belt motif also mediates the assembly and disassembly of the HORMA-domain protein dimers (44, 45). HORMA domains can adopt an open and a closed state (40). The closed state has two β-strands in the safety belt moving away from its core and wrapping around the HORMA domain, allowing HORMA proteins to bind their partners (Fig. 3, *C* and *F*). Our apo Polζ structure shows that the safety belts of Rev7A and Rev7B adopt a closed conformation in their Rev3^RBM1^-and Rev3^RBM2^-bound states, respectively (Fig. 3*F*). The yeast Rev7 dimer also shows a head-to-tail arrangement when bound to the RBM1 and RBM2 motifs of Rev3 (Fig. 3, *A* and *B*).

### Binding modes of yeast and human Rev7

Budding yeast and human Rev7 only share 27% amino acid sequence identity (Fig. S8*A*) but comparison of their 3D structures shows similar folds. The structure of human Polζ is still unavailable, but we examined the crystal structures of human Rev7 in complex with fragments of shieldin-3 (SHLD3) (46, 47, 48), including that with a SHLD3 peptide (41–74 aa), which we determined to a resolution of 2.0 Å (Table 2) to compare the binding modes of yeast and human Rev7. SHLD3 and Rev7 are two of the four components of the protein complex shieldin that plays a key regulatory role in DNA double-strand break repair by blocking DNA end resection necessary for homologous recombination (49, 50, 51). The small backbone RMSDs with respect to Rev7A-Rev3^RBM1^ (RMSD 1.20 Å) and Rev7B-Rev3^RBM2^ (RMSD 1.08 Å) indicate that the yeast and human Rev7 folds are very similar. In human just like in yeast, Rev7 forms a homodimer, but the orientations of the two Rev7 protomers differ radically in the two systems (Fig. 3, *A* and *B*). In addition, the safety-belt region of yeast Rev7 is longer than that of human Rev7. In yeast Rev7, the safety belt presents a short helix-turn-helix motif, but the one in human Rev7 is a short helix connected to a loop (Fig. S8*B*). Since the two yeast Rev7 molecules have different organizations in the Polζ complex, we compared each to our human Rev7-SHLD3 crystal structure, which was determined in a Rev7 monomeric state using the R124A mutation (Fig. 3*C*). We superimposed the Rev7 structure in human Rev7(R124A)-SHLD3 to the Rev7 structures in yeast Rev7A-Rev3^RBM1^ and Rev7B-Rev3^RBM2^. The binding modes are very different (Fig. 3). Rev7B W177 and V178 interact with W604 and Y606 of Rev3^RBM2^ through hydrophobic interactions or π-stacking contacts (Fig. 3*G*), while Rev7A D137 forms a weak hydrogen bond with Q531 of Rev3^RBM1^ (Fig. 3*H*). In the crystal structure of Rev7(R124A)-SHLD3, Rev7 binds the SHLD3 peptide at two sites. In one site, SHLD3 N-terminal loop residues P50 and L51 interact mainly *via* hydrophobic interactions with Rev7 residues A174, M160, and L173 in the safety-belt region of Rev7 (Fig. 3*D*). In another hydrophobic interface, residues I60 and A65 in the C-terminal α-helix (αC-helix) of SHLD3 interact with Rev7 Y37 and P38 (Fig. 3*E*). As can be seen from side-by-side comparison (Fig. 3, *B*, *C* and *F* and Fig. S8*A*), yeast and human Rev7 show little similarity in target recognition. Therefore, one may not reliably infer from the yeast Polζ structure how human Rev7 binds other components of the human Polζ complex.

**Table 2:** Data collection and refinement statistics for human Rev7(R124A)-SHLD3 (41–74 aa)

Protein	Human Rev7(R124A)-SHLD3 (41–74 aa)
Data collection	
Space group	P32 2 1
Cell dimensions	
*a*, *b*, *c* (Å)	59.99, 59.99, 132.47
α, β, γ (°)	90, 90, 120
Resolution (Å)	29.25–2.00 (2.05–2.00)^a^
*R*_sym_ or *R*_merge_	0.083 (1.193)
*I*/σ*I*	40.89 (4.11)
Completeness (%)	98.40 (98.89)
Redundancy	22.6 (20.9)
Refinement	
Resolution (Å)	29.25–2.00 (2.05–2.00)
No. reflections	19,167 (1339)
*R*_work_/*R*_free_	0.22 (0.30)/0.24 (0.32)
No. atoms	2016
Protein	1817
Ligand/ion	15
Water	184
*B*-factors	
Protein	36.17
Ligand/ion	114.28
Water	45.14
R.m.s. deviations	
Bond lengths (Å)	0.004
Bond angles (°)	0.65
Ramachandran plot (%)	
Disallowed	0.00
Allowed	0.44
Favored	99.56

a Values in parentheses are for highest-resolution shell.

## Discussion

In this paper, we have determined the structure of the apo form of budding yeast DNA polymerase Polζ, revealing a closed conformation in the absence of DNA. DNA binding to Polζ induces a concerted movement of local structural motifs associated with the opening of the central DNA-binding channel of the polymerase. Moreover, we identified a lysine (K1214) in Rev3 as a putative gating residue for DNA binding. In our apo Polζ structure, the side chain of K1214 protrudes out and obstructs the DNA-binding channel. In the presence of DNA, the side chain of K1214 moves away from the central channel and creates a wider space that allows insertion of incoming DNA. Future molecular dynamics simulations will incorporate this new knowledge to further explore the mechanism of action of yeast Polζ.

The Rev7 subunit of Polζ is an evolutionarily conserved hub protein found in many different protein complexes. For example, human Rev7 is not only a component of Polζ but is also an essential subunit of DNA double-strand break repair protein complex shieldin. Since there is no structure of human Polζ, we compared the molecular interactions of human Rev7 in the context of shieldin to those of Rev7 in yeast Polζ. Although the yeast and human Rev7 proteins display virtually identical folds, their dimeric conformations and how they bind target proteins differ markedly. These differences are surprising and suggest that the oligomeric conformation of Rev7 may be context-dependent if one assumes that the spatial arrangement of subunits is the same in budding yeast and human Polζ. Alternatively, yeast and human Polζ may differ in structure and modes of action. A future challenge will be to determine the structure of human Polζ, which would be of high value for the long-term development of new anticancer drugs (15, 17, 52, 53).

## Experimental procedures

### Yeast cells and plasmids

Protease-deficient yeast (*S. cerevisiae*) host PY265 (PY265, mat a, genotype: can 1 his3 leu 2 trp 1 ura 3 pep4::HIS3 GAL nam7Δ::Mx4) and plasmids pBL813-Zeta_opt (for expression of GST-Rev3 and Rev7 under the control of a GAL1-10 promoter with codon-optimized genes) (URA selection), pBL347_p31-32_his (for expression of Pol31 and heptahistidine-Pol32 under the control of a GAL1-10 promotor) (LEU selection), and pBL824-0 Rev1 PPCS (for expression of yeast GST-Rev1 under the control of a GAL1-10 promotor) (URA selection) were gifts from Dr P.M. Burgers (13) (Washington University, Saint Louis, MO).

### Cloning of human Rev7 and SHLD3

The cDNAs of the full-length human Rev7 and various lengths of shieldin-3 (SHLD3) (full-length, 1–29 aa, 28–74 aa, 41–74 aa, 1–83 aa, and 28–83 aa) were dually inserted into a pETDuet1-based vector, producing coexpressed Rev7-SHLD3 complexes in which Rev7 has an N-terminal hexahistine tag cleavable by PreScission protease and SHLD3 is untagged. QuikChange (Agilent) was used to introduce a homodimer disrupting R124A single point mutation in Rev7.

### Yeast transformation

Plasmids were amplified by chemical transformation of TOP10 cells *E coli* (Thermo Fisher Life Technologies) with ampicillin selection. Plasmids (pBL813-Zeta_opt, pBL347_p31.32_his and pBL824-0 Rev1 PPCS) were purified using Wizard Plus DNA purification kits (Promega) and then used for PY265 yeast transformation by electroporation.

### Expression of full-length yeast Polζ

A single colony transformed with pBL813-Zeta_opt and pBL347_p31.32_his plasmids from an SD-Ura-Leu agarose plate (Synthetic Defined (SD) agar plates: 2% glucose, 6.7 g yeast nitrogen base without amino acids, 20 mg each of histidine, arginine, tryptophan, tyrosine, methionine and adenine, 40 mg threonine, 50 mg phenylalanine, 60 mg lysine and 20 g agar in 1 l) was used to inoculate 30 ml of SD-Ura-Leu media (SD components without agar). The culture was grown for 3 days at 30 °C and 240 rpm to achieve cell saturation. Once sufficiently dense, a primary SCGL starter culture was created by inoculating 30 ml of SCGL-Ura-Leu media with 500 μl of cells grown in SD media. The SCGL medium contains per liter: 1.7 g of yeast nitrogen base without amino acids and ammonium sulfate, 5 g ammonium sulfate, 30 ml glycerol, 20 ml lactic acid, 1 g glucose, 20 mg each of adenine, histidine, tryptophan, proline, arginine, and methionine, 30 mg each of isoleucine, tyrosine, and lysine, 50 mg phenylalanine, and 100 mg each of glutamic acid, aspartic acid, valine, threonine, and serine. Uracil and leucine were omitted to ensure the selective maintenance of plasmids. Prior to autoclaving, the pH of the media was adjusted to 5-6. The primary starter was grown for 2 to 3 days at 30 °C and 240 rpm. Secondary cultures were produced by filling 50 ml conical tubes with 30 ml of SCGL-Ura-Leu media and inoculating each with 1 ml of the primary starter. Secondary cultures were grown for 2 to 3 days at 30 °C and 240 rpm and then used to inoculate SCGL-Ura-Leu media in 2 l baffled Erlenmeyer flasks (50 ml starter per 600 ml media). Growth was continued at 30 °C and 240 rpm for 24 to 26 h to achieve an OD_660_ of 2.8 to 3.0. At this point, an equal volume of rich YPGLA medium (30 g yeast extract, 60 g peptone, 90 ml glycerol, 60 ml lactic acid and 60 mg adenine per l, pH adjusted to 5–6) was added to each flask. The flasks were gently swirled and then the mixed media distributed equally between two 2 l flasks. Cells were grown for 2 to 3 h at 30 °C and 240 rpm and then induced with 2% solid galactose. Cell growth was continued for 12 to 16 h. Cells were harvested by centrifugation at 7000*g* for 10 min at 4 °C using a swinging bucket rotor in a Lynx 4000 centrifuge. To avoid disruption of the cell pellet, rotor deceleration was set to a low level of 4. Centrifuged cells were washed with ice cold water and centrifuged again. The resulting pellet was transferred to sterile 50 ml conical tubes and immediately frozen using liquid nitrogen for storage at −80 °C.

### Purification of Polζ

All steps were performed at 4 °C unless otherwise stated. Frozen cells in which the four subunits of Polζ were expressed were thawed using room temperature water and then transferred to a bead beater chamber for lysis. To 200 g yeast cells in the chamber, were added 100 ml of lysis buffer (150 mM HEPES (pH 7.8), 900 mM KCl, 90 mM K_2_HPO_4_/HK_2_PO_4_, 8% glycerol, 7.5 mM sucrose, 0.15% Tween 20, 0.03% Nonidet P-40, 6 mM DTT, 30 μM pepstatin A, 30 μM leupeptin, 7.5 mM benzamidine, and 0.5 mM PMSF), and 150 ml of 0.5 mm glass beads. Cells were lysed by 55 cycles of alternating lysis (30 s) and cooling (2 min). After lysis, the homogenate was removed from the chamber and transferred to a clean beaker. The glass beads were allowed to settle, the supernatant was collected, and the beads were rinsed three times with lysis buffer to ensure collection of the entire homogenate. Nucleic acids were precipitated from the homogenate by adding 45 ml of 10% Polymin P per l of homogenate. The mixture was stirred for 20 min and then centrifuged at 29,000*g* for 60 min to remove cellular debris/insoluble material. The supernatant was collected, and ammonium sulfate was added to a concentration of 0.31 g/ml. The sample was stirred overnight and then centrifuged at 29,000*g* for 90 min. The resulting pellet was collected and resuspended in Buffer A1 (50 mM HEPES (pH 7.4), 300 mM KCl, 30 mM K_2_HPO_4_/KH_2_PO_4_, 8% glycerol, 2.5 mM sucrose, 0.05% Tween 20, 0.01% Nonidet P-40, 2 mM DTT, 8 μM pepstatin A, 8 μM leupeptin, 2 mM benzamidine, and 0.5 mM PMSF) in a total volume of 1 l for 1 h before centrifuging at 29,000*g* for 5 h. The resulting soluble material was collected and filtered using a 0.45-μm bottle top filter. The filtered material was passed over a GST-Prep FF 16/10 affinity column (GE Healthcare/Cytiva) at a flow rate of 2 ml/min using a peristaltic pump. For batch chromatography, protein pellet was resuspended with 3 l Buffer A1 for 12 to 16 h, centrifuged at 29,000*g* for 2 h, incubated with 20 ml glutathione-agarose resin (Thermo Fisher Scientific) for 4 h, and then packed into a disposable column. Following application of the supernatant material, the column was washed with 200 ml each of Buffer A2 (30 mM HEPES (pH 7.8), 200 mM KCl, 30 mM K_2_HPO_4_/KH_2_PO_4_, 8% glycerol, 2.5 mM sucrose, 0.05% Tween 20, 0.01% Nonidet P-40, 1 mM DTT, 5 mM MgCl_2_, 1 mM ATP, 2 μM pepstatin A, and 0.5 mM PMSF), and Buffer A3 (30 mM HEPES (pH 8.0), 100 mM KCl, 30 mM K_2_HPO_4_/KH_2_PO_4_, 8% glycerol, 2.5 mM sucrose, 0.05% Tween 20, 0.01% Nonidet P-40, 1 mM DTT, 2 μM pepstatin A, and 0.5 mM PMSF) at a flow rate of 2.5 to 3.0 ml/min. The Polζ complex was eluted from the resin using Buffer A3 with 50 mM reduced glutathione, at a flow rate of 0.5 ml/min. Three milliliter fractions were collected and analyzed by SDS-PAGE. The Polζ complex-containing fractions were combined and treated with PreScission protease (Cytiva) to cleave the GST tag from Rev3. The sample was then diluted with equal volume of Buffer E (30 mM HEPES (pH 7.4) 20 mM KCl, 20 mM K_2_HPO_4_/KH_2_PO_4_, 5% glycerol, 2.5 mM sucrose, 1 mM DTT, and 0.5 mM PMSF), and imidazole was added to a final concentration of 20 mM. The sample was applied to a HisPrep FF 16/10 nickel affinity column (Cytiva) at a flow rate of 3 ml/min using a peristaltic pump. The column was washed with 100 ml of Buffer E followed by 100 ml of Buffer E containing 20 mM imidazole at a flow rate of 3 ml/min. Polζ was eluted from the column with Buffer E containing 200 mM imidazole at a flow rate of 0.5 ml/min. Fractions eluted from the column were analyzed by SDS-PAGE and gels were stained with Coomassie Blue or silver. The four components of the Polζ complex were analyzed by mass spectrometry. When performing bulk chromatography, fractions treated with PreScission protease were diluted 5× with Buffer A3 with 400 mM KCl and 20 mM imidazole. Two milliliter of Ni-Sepharose 6 resin (GE Healthcare) was added to the diluted sample, stirred for 2 h, and packed into a disposable column. The resin was washed with 100 ml of Buffer A3 with 400 mM KCl and 20 mM imidazole, and the protein was eluted using Buffer A3 with 400 mM KCl and 200 mM imidazole.

### Purification of Rev1

Full-length GST-Rev1 was purified from ∼180 g of Rev1 expressing yeast cells following the same steps used for the purification of the Polζ complex but without the nickel affinity chromatography step.

### Purification of human Rev7 and SHLD3

Various Rev7(R124A)-SHLD3 complexes were coexpressed in BL21(DE3) *E. coli* cells grown at 37 °C in LB media to an OD_600_ of ∼0.6 and induced with 0.5 mM isopropyl-β-D-thiogalactoside at 15 °C for ∼16 h. Harvested cells were resuspended in bind buffer, lyzed with a microfluidizer (Avestin Emulsiflex C5), and centrifuged. The resulting supernatant was loaded onto a column with Ni^2+^-NTA agarose (Qiagen). After extensively washing the column with wash buffer, the complex was eluted with elution buffer. The bind buffer is made up of 50 mM sodium phosphate (pH 7.5) and 300 mM NaCl, while the wash and elution buffers have the bind buffer components with additional 20 and 250 mM imidazole, respectively. The hexahistidine tag on Rev7(R124A) was removed by addition of PreScission protease at 4 °C overnight. The complex was further purified by size-exclusion chromatography using a HiLoad 16/60 Superdex 75 column (GE Healthcare) and 5 mM HEPES (pH 7.4) and 100 mM NaCl as running buffer. From protein expression results, a minimal complex of Rev7(R124A) and SHLD3 (41–74 aa) could be formed. This complex was used for structure determination.

### Mass spectrometry of Polζ

Gel bands obtained from SDS-PAGE gels were subjected to in-gel trypsin digestion after reduction and carboxymethylation, and the treated and extracted peptides were analyzed by nano-ESI-LC/MS/MS with a Q Exactive mass spectrometer coupled to a Dionex nano-LC system (Thermo Fisher Scientific). The LC system used multistep linear gradients with solvents A (2% acetonitrile, 0.2% formic acid, in water) and B (80% acetonitrile, 10% isopropyl alcohol, 0.2% formic acid, in water) as follows: 4 to 5 min, at 5% B; 5 to 35 min 5 to 45% B; 35 to 38 min 45 to 95% B; 38 to 42 min 95% B; 42 to 44 min 95% A–10% B; 44 to 47 min 10% B; 47 to 55 min 10 to 95% B; 55 to 58 min 95% B; 58 to 61 min 95 to 5% B; 61 to 67 min 5% B. The mass spectrometer had a resolution of 70,000 (at 200 m/z) and used data dependent acquisition, with a full MS1 scan ranging from 350 to 1800 m/z, then selecting the top 15 ions for MS2 analysis with a dynamic range set to 8 s. All MS/MS spectra were analyzed using Mascot (version 2.4; Matrix Science), and X! Tandem (www.thegpm.org; version 2013.09.01 is provided in the public domain by the Global Proteome Machine Organization, Manitoba Centre for Proteomics and Systems Biology). Each software was set up to search the current SwissProt database, assuming trypsin digestion with up to two miscleavages with a fragment ion tolerance of 10.0 PPM (www.uniprot.org; SwissProt). Oxidation of methionine was set as a variable modification, and carbamidomethylation of cysteine (iodoacetamide derivative) was set as a fixed modification. Proteomics software (Scaffold, ver. 4.11.0; Proteome Software Inc) was used to view MS/MS-based peptide and protein identifications. Peptide identifications were accepted if they could be established at > 95.0% probability, as specified by the peptide prophet algorithm. Protein identifications were accepted if they could be established at > 95% probability and contain at least two unique peptides. Protein probabilities were assigned by the protein prophet algorithm.

### Database searching for Polζ

Tandem mass spectra were extracted using ProteoWizard MsConvert. Charge state deconvolution and deisotoping were not performed. All MS/MS samples were analyzed using Mascot (Matrix Science; version 2.4.0) and X! Tandem (The GPM, thegpm.org; version X! Tandem Sledgehammer (2013.09.01.1)). Mascot was set up to search the *S. cerevisiae* Swissprot database (downloaded in April 2019, 16,060 entries), assuming the digestion enzyme stricttrypsin. X! Tandem was set up to search the *S. cerevisiae* Swissprot database (downloaded in April 2019, 16,060 entries) also assuming stricttrypsin. Mascot and X! Tandem were searched with a fragment ion mass tolerance of 0.020 Da and a parent ion tolerance of 10.0 ppm. Carbamidomethyl of cysteine was specified in Mascot and X! Tandem as a fixed modification. Glu->pyro-Glu of the N-terminus, ammonia-loss of the N-terminus, Gln->pyro-Glu of the N-terminus, and oxidation of methionine were specified in X! Tandem as variable modifications. Oxidation of methionine and acetyl of the N-terminus were specified in Mascot as variable modifications.

### Criteria for Polζ identification

Scaffold (version Scaffold_4.11.0, Proteome Software Inc) was used to validate MS/MS-based peptide and protein identifications. Peptide identifications were accepted if they could be established at ≥95.0% probability by the Scaffold Local FDR algorithm. Protein identifications were accepted if they could be established at ≥95.0% probability and contained at least two identified peptides. Protein probabilities were assigned by the Protein Prophet algorithm (54). Proteins that contained similar peptides and could not be differentiated based on MS/MS analysis alone were grouped to satisfy the principles of parsimony. Proteins sharing significant peptide evidence were grouped into clusters.

### Database searching for Rev1

Tandem mass spectra were extracted using ProteoWizard MsConvert. Charge state deconvolution and deisotoping were not performed. All MS/MS samples were analyzed using Mascot (Matrix Science; version 2.4.0). Mascot was set up to search the Swissprot *S. cerevisiae* database (downloaded in August 2019, 13,582 entries), assuming the digestion enzyme stricttrypsin. Mascot was set up to search with a fragment ion mass tolerance of 0.020 Da and a parent ion tolerance of 10.0 ppm. Carbamidomethyl of cysteine was specified in Mascot as a fixed modification. Oxidation of methionine was specified in Mascot as a variable modification.

### Criteria for Rev1 identification

Scaffold (version Scaffold_4.11.0, Proteome Software Inc) was used to validate MS/MS-based peptide and protein identifications. Peptide identifications were accepted if they could be established at ≥95.0% probability. Peptide probabilities from Mascot (samples (qe1_2020feb21_P20025_upperband) and (qe1_2020feb21_P20025_lowerband)) were assigned by the Scaffold Local FDR algorithm. Peptide probabilities from Mascot (samples (qe1_2020feb21_P20025_postrunblank) and (qe1_2020feb21_P20025_prerunblank)) were assigned by the PeptideProphet algorithm (55) with Scaffold delta-mass correction. Protein identifications were accepted if they could be established at ≥95.0% probability and contained at least two identified peptides. Protein probabilities were assigned by the ProteinProphet algorithm (54). Proteins that contained similar peptides and could not be differentiated based on MS/MS analysis alone were grouped to satisfy the principles of parsimony. Proteins sharing significant peptide evidence were grouped into clusters.

### Biolayer interferometry and K_D_ determination

Biolayer interferometry assays were performed on a BLItz biolayer interferometry instrument (ForteBio). All measurements were done at 22 °C in basic kinetic mode and consisted of three main steps. An initial base line reading (30 s) using 1× kinetic buffer (10× kinetic buffer, #18-1105 Pall ForteBio was diluted to 1× with PBS, #10010-23 Gibco) was performed. This was followed by a step in which association of proteins occurred over 600 s. A dissociation step of 120 s was then performed. Prior to use, Ni^+2^-NTA sensor tips (Pall ForteBio) were equilibrated in 1× kinetic buffer for at least 20 min. Yeast Polζ (19 μg/ml, 328 kDa) (Rev3 with a GST-tag, heptahistidine--tagged Rev7, Pol31, and Pol32) in Buffer E with 50 mM glutathione was diluted with 1× kinetic buffer to 9.5 μg/ml for association with Ni^+2^-NTA tips on the Blitz. Yeast Rev1 (142 μg/ml, 112 kDa) in Buffer A3 with 50 mM glutathione was diluted 30-fold with 1× kinetic buffer to 4.7 μg Rev1/ml and likewise applied to Ni^+2^-NTA tips.

### Translesion DNA polymerase assay

Extension of a 32-base DNA oligonucleotide primer annealed to a 52-base DNA oligonucleotide containing an abasic site (tetrahydrofuran covalent linked dSpacer in oligonucleotide sugar backbone, without a purine or pyrimidine base) was prepared to test translesion extension ability of Polζ and Rev1. Below are sequences of the synthetic oligonucleotides (GENEWIZ).

32-mer:

5′-GTTTTCCCAGTCACGACGATGCTCCGGTACTC-3′

52-mer:

5′-TTCGTATAATGCCTACACT∗GAGTACCGGAGCATCGTCGTGACTGGGAAAAC-3′

(∗ = abasic site: tetrahydrofuran without nucleotide base)

The 32-base primer was first end labeled with ^32^P using gamma ^32^P ATP (adenosine 5′-triphosphate, #BLU-002H PerkinElmer) and T4 polynucleotide kinase (New England Biolabs) at 37 °C in T4 polynucleotide kinase buffer (70 mM Tris-HCl (pH 7.6), 10 mM MgCl_2_, and 5 mM DTT). After labeling, sample was passed through a Micro Bio-Spin P6 spin column (Bio-Rad) pre-equilibrated with annealing buffer (10 mM Tris-HCl (pH 7.6), 50 mM NaCl and 1 mM EDTA) to remove excess ^32^P ATP. The ^32^P labeled 32-base primer and the unlabeled 52-base template were annealed by putting the tube with the mixture in a 500 ml beaker of boiling water and letting the water cool to room temperature for ∼4 h. The annealed DNA was stored at −20 °C until use.

Ten microliter solution of 100 fmol ^32^P-labeled annealed DNA in 1× translesion DNA extension buffer (25 mM KH_2_PO4 (pH 7.0), 5 mM MgCl_2_, 5 mM DTT, 100 μg/ml BSA, and 10% glycerol) (56) was added to each of the mixtures below.

To test TLS DNA polymerase activity of Polζ, mixtures containing 53, 131, or 526 fmol of Polζ in 20 μl Buffer A3 with 25 mM glutathione were prepared. To examine enhancement of Polζ activity by Rev1, a similar 20 μl mixture containing 53 fmol of Polζ and 12 fmol of GST-Rev1 was prepared. Mixtures containing only 12, 30, and 121 fmol of Rev1 in 20 μl were likewise set up to examine the polymerase activity of Rev1. After combining DNA and protein mixtures on ice, DNA synthesis (translesion and extension) was initiated by addition of 0.1 mM deoxynucleotide triphosphates (dNTPs) and incubation at 30 °C. The reactions were stopped after 30 min by addition of 10 μl 1× Novex Hi-Density TBE sample buffer (Thermo Fisher Scientific). Reaction samples were stored at −20 °C or combined with equal volume of 80% formamide, 1× TBE. Samples from the latter were heated to 95 °C for 6 min, loaded onto 1× TBE 7 M urea 10% acrylamide gels (Thermo Fisher Scientific) prewarmed to 45 °C and with buffer prewarmed to 50 °C, and run at 180 V constant voltage until loading dye was near bottom of gel (∼q20 bp). ^32^P-labeled 32-mer primer and ^32^P-labeled 52-mer template (150 fmol each in 1× NOVEX Hi-Density TBE sample buffer), prepared similarly as the samples, were also run on gels to serve as molecular weight standards. Undried gels covered with plastic wrap were exposed to X-ray films for various lengths of time (h-days) and developed.

### X-ray crystallography of human Rev7 and SHLD3

Crystals of the full-length Rev7(R124A)-SHLD3 (41–74 aa) were obtained by the hanging drop method, putting 1 μl of the protein sample (25 mg/ml in 5 mM HEPES (pH 7.4), 100 mM NaCl, and 5 mM DTT) and 1 μl of the reservoir solution for the drop and 0.5 ml for the reservoir solution (0.1 M MES monohydrate (pH 6.5) and 1.4 M MgSO_4_·6H_2_O) in the well. Crystals formed within 2 to 4 weeks at 15 °C. The crystals were cryoprotected in 50% PEG400 and quick-frozen in a cryoloop with liquid nitrogen. Diffraction data were collected at the 19-BM beamline at the Advanced Photon Source, Argonne National Laboratory. Diffraction patterns were indexed, integrated, and scaled with HKL2000 (57). The initial phases were obtained by molecular replacement using the coordinates of the Rev7-Rev3 structure (PDB code: 3ABD) as a search model in Phenix (36, 58). The starting model was completed and refined in Coot (59) and Phenix (58) in an iterative manner. The crystals of Rev7 (R124A)-SHLD3 (41–74 aa) complex have a P3_2_21 space group, with one molecule of Rev7(R124A) and one molecule of SHLD3 (41–74 aa) in the complex. One copy of the complex molecule is found in the asymmetric unit. Statistics of the final structure are shown in Table 2. All molecular representations were generated with PyMOL (http://www.pymol.org) and UCSF Chimera (60).

### Negative-stain electron microscopy

Negatively stained samples of 0.01 mg/ml apo Polζ complex were prepared using 0.75% uranyl formate and followed by the previous method (61). The stained samples were imaged using a Tecnai TF20 TEM at an accelerating voltage of 200 keV with a CCD camera recording at a pixel size of 1.4 Å/pixel at the specimen level. Thirty-nine electron images were collected and imported into Relion (version 3.1-beta-commit-ca101f) (62) for general image processing. A total of 2330 particles were manually selected from the electron images, and the 2D class averages with an assigned *k* of 50 were calculated, respectively.

### Single-particle cryo-EM data collection

The Polζ complex sample was loaded onto a Superose 6 column (GE Healthcare) for size-exclusion chromatography. The purified peak fraction was used for further cryo-EM imaging. A C-flat 400-mesh holey-carbon-coated copper grid (2/1 4C; Protochips) was glow-discharged for 15 s in a Pelco easiGlow glow-discharge system (Ted Pella). Five microliter of 0.1 mg/ml protein sample was then applied on the EM grid. The grid was blotted by a filter paper to remove the excess solution and quickly frozen into liquid ethane. The plunge freezing process was automated using a Vitrobot Mark IV plunge freezer (Thermo Fisher/FEI) at a humidity of 100% with a blotting time of 6 s.

All the cryo-EM data collections were completed in the Eyring Materials Center (EMC) at Arizona State University (ASU). The grid specimen was imaged using a Thermo Fisher/FEI Titan Krios TEM (Thermo Fisher/FEI) at an accelerating voltage of 300 keV. The electron scattering was recorded by a Gatan Summit K2 direct electron detector (DED) camera in superresolution mode (63). The nominal magnification was set to 48,780×, corresponding to a pixel size of 1.025 Å/pixel at the specimen level. The defocus was set to vary from −0.8 to −3.0 μm. The camera counted rate was calibrated to 8 e^−^/pixel/s. The exposure time was 6 s, accumulating to a total dosage of 45.7 e^−^/Å^2^. The dataset was collected in counting mode. The beam-image shift scheme was applied to accelerate the data collection (64). The procedure of low-dose imaging was automated using SerialEM software (version 3.9) with customized macros (65).

### Image processing

Image processing was generally conducted using cryoSPARC (version 3.0) (66). A total of 11,698 cryo-EM movies was imported into the program for processing. The frame registration and averaging for motion correction were performed using the “Patch motion correction” and the estimation of the defocus was performed using the “Patch CTF estimation.” An ensemble of 2,974,553 particles was automatically selected using a neural network and positive-unlabeled learning by the Topaz program (version 0.2.3) (67). The curation of the particle images was performed using iterative 2D image classification. A total of 1,658,585 particles was selected for an *ab initio* 3D map generation (66). The two generated volumes were refined further against their individual particle subsets and only one volume showed discernible structural features of a protein. The subgroup of its 3D reconstruction with discernible protein features was carried over for homogeneous refinement and subsequent image processing. A total of 213,120 particle images was selected for further processing. The 3D map was then refined using homogeneous and nonuniform refinement procedures in cryoSPARC (66, 68). The final map resolution reached 4.11 Å, estimated using the golden standard FSC method at the cutoff of 0.143 (29). The local resolution was assessed using an FSC-windowed method (69). The directional FSC of the reconstruction was assessed using 3DFSC program wrapped in cryoSPARC (70).

Further signal subtraction and focused classification were performed to improve the quality of the local densities for Rev3 and Rev7-Pol31-Pol32 using cryoSPARC software (66). Masks were generated using Segger implemented in UCSF Chimera (71). The local refinement was focused on the region of interest and the remaining densities were subtracted (30). The resolutions of the local densities of the Rev3 and Rev7-Pol31-Pol32 were 3.65 Å and 3.72 Å, respectively. The two improved maps were then combined using the “phenix.combine_focused_maps” program in Phenix software (version 1.18.2-3874) for subsequent modeling (58).

### Molecular modeling

Previous atomic coordinates of the Polζ-DNA complex (for which DNA density was not detected) (PDB code: 6V8P) (28) were used as starting template. These initial coordinates were first docked into the cryo-EM density using the “Fit in the Map” function in UCSF Chimera software (version 1.14) (60). The fitted coordinates were manually rebuilt and adjusted using Coot (version 0.9-pre) (59, 72). The rebuilt coordinates were refined against the cryo-EM density using the “phenix.real_space_refine” program in Phenix software package (version 1.18.2-3874) (58). The molecular graphic presentation for the final model was made using UCSF Chimera or UCSF ChimeraX (version 0.91) (73).

The structures of the DNA-bound (PDB code: 6V8P and 6V93) (28) and apo Polζ were used to calculate a morph movie by using the Needleman–Wunsch algorithm in UCSF Chimera (60). The generated movie is shown in Movie S1.

## Data availability

Further information and requests for resources and reagents should be directed to and will be fulfilled by the corresponding authors. There is no restriction on materials generated for this study and first reported here. The accession numbers for the data reported in this paper are PDB: 7LXD, EMDB: EMD-23570 (yeast Polζ complex), and PDB: 6VE5 (human full-length Rev7(R124A)-SHLD3 (41–74 aa)). The mass spectrometry data of Polζ and Rev1 can be accessed in the MassIVE database under accession codes MSV000087408 and MSV000087410. All other data are available from the corresponding authors upon request.

## Supporting information

This article contains supporting information.

## Conflict of interest

The authors declare that they have no conflicts of interest with the contents of this article.
