# Supplementary material for: Cryo-EM reveals conformational flexibility in apo DNA polymerase ζ
Source: J Biol Chem. 2021 Jun 24;297(2):100912. doi: 10.1016/j.jbc.2021.100912 (PMC8319531; doi:10.1016/j.jbc.2021.100912)
Supplement: Movie S1 [file mmc2.pdf]

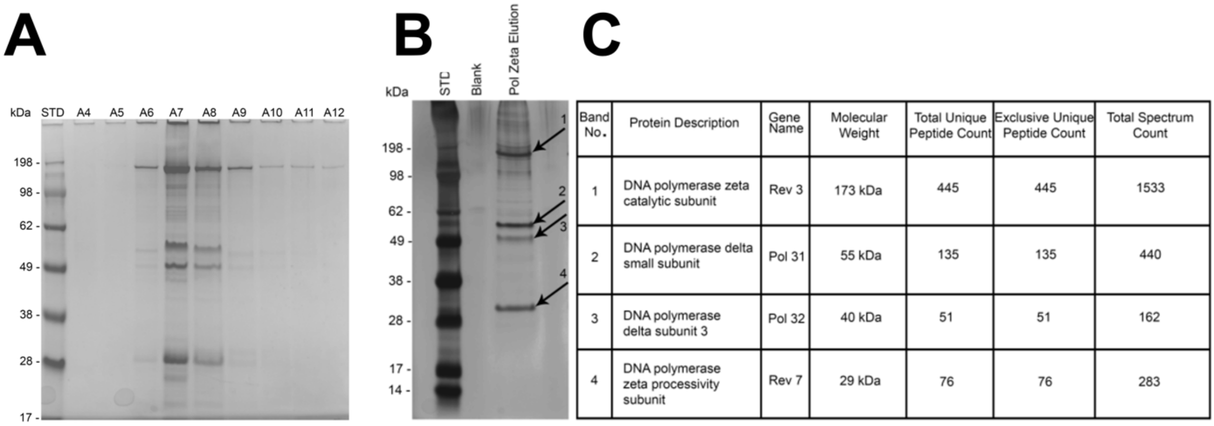

C

Band No.	Protein Description	Gene Name	Molecular Weight	Total Unique Peptide Count	Exclusive Unique Peptide Count	Total Spectrum Count
1	DNA polymerase zeta catalytic subunit	Rev 3	173 kDa	445	445	1533
2	DNA polymerase delta small subunit	Pol 31	55 kDa	135	135	440
3	DNA polymerase delta subunit 3	Pol 32	40 kDa	51	51	162
4	DNA polymerase zeta processivity subunit	Rev 7	29 kDa	76	76	283

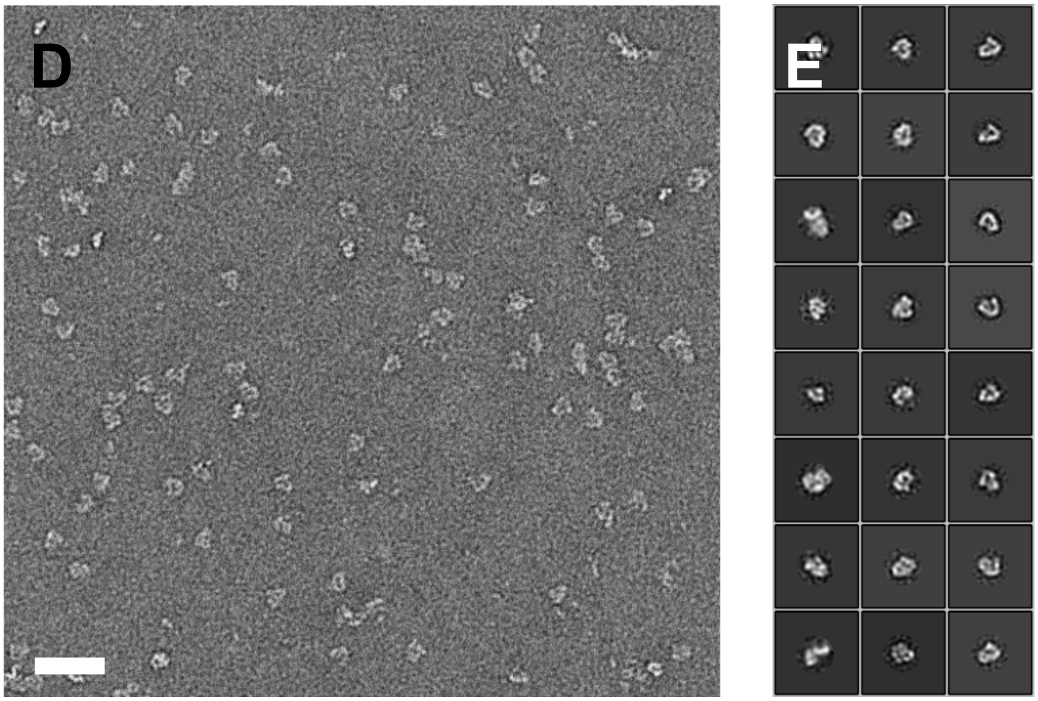

Figure S1. Purification and analysis of *Saccharomyces cerevisiae* Polζ. (A) Coomassie blue stained SDS-PAGE gel of fractions eluted from a nickel-affinity column. Note the presence of prominent bands at 175, 55, 50 and 28 kDa in lanes A7 and A8. **(B)** Silver-stained SDS-PAGE gel of Polζ complex elution. **(C)** Summary of the mass spectrometry results for the eluted Polζ complex. Total unique peptide count indicates the number of different amino acid sequences that are associated with the protein that are unique to that protein. Exclusive unique peptide count represents the number of different amino acid sequences, regardless of any modification, which are associated with a single protein group. Total spectrum count is the number of unique spectra associated with each protein including those shared with other proteins. **(D)** Electron micrograph of negatively stained apo Polζ. White contrasts are Polζ complexes in a black background. Scale bar indicates 40 nm. **(E)** Representative 2D class averages of the protein complexes. Box side length is 560 Å.

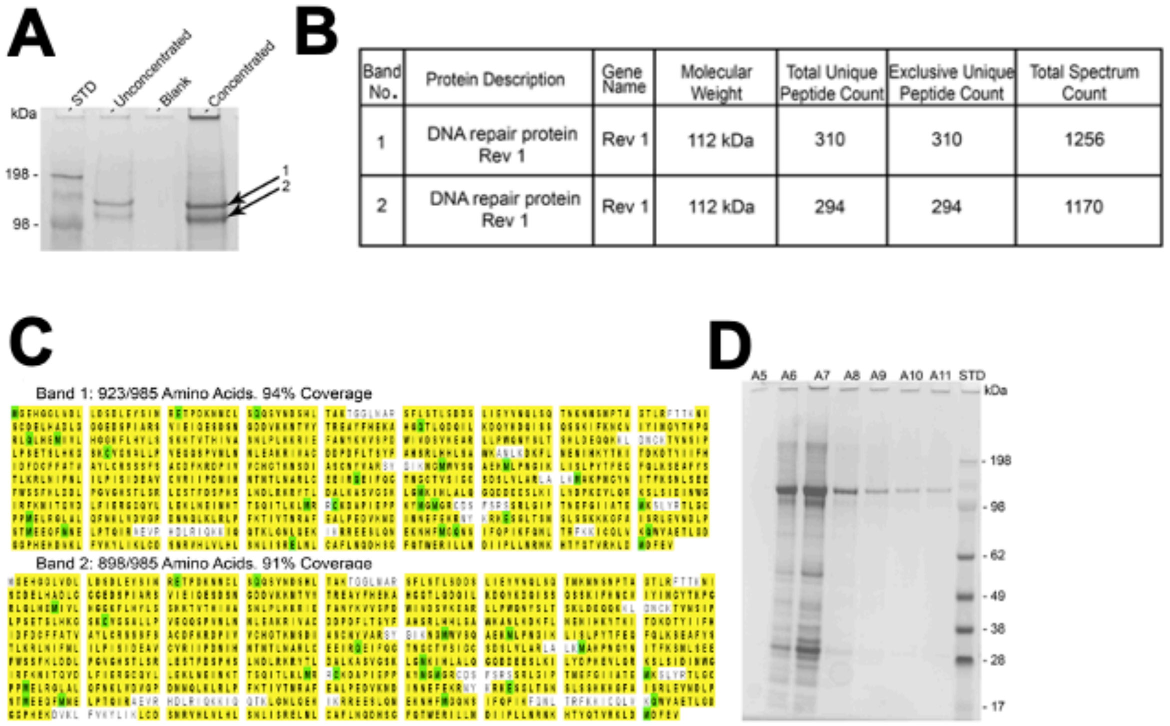

Band No.	Protein Description	Gene Name	Molecular Weight	Total Unique Peptide Count	Exclusive Unique Peptide Count	Total Spectrum Count
1	DNA repair protein Rev 1	Rev 1	112 kDa	310	310	1256
2	DNA repair protein Rev 1	Rev 1	112 kDa	294	294	1170

Figure S2. Analysis of purified full-length *Saccharomyces cerevisiae* Rev1. (A) Full-length yeast Rev1 elution analyzed by SDS-PAGE and silver staining. Indicated gel bands were analyzed by mass spectrometry. **(B)** Condensed mass spectrometry results of eluted Rev1 doublet. **(C)** Sequence coverage for each of the two bands that were identified as Rev1. Green highlights are identified residues modified in sample preparation or source fragmentation in mass spectrometry measurements. **(D)** Purification of yeast Rev1. SDS-PAGE and Coomassie Blue staining of full-length Rev1 elution from GST (glutathione S-transferase)-affinity chromatography.

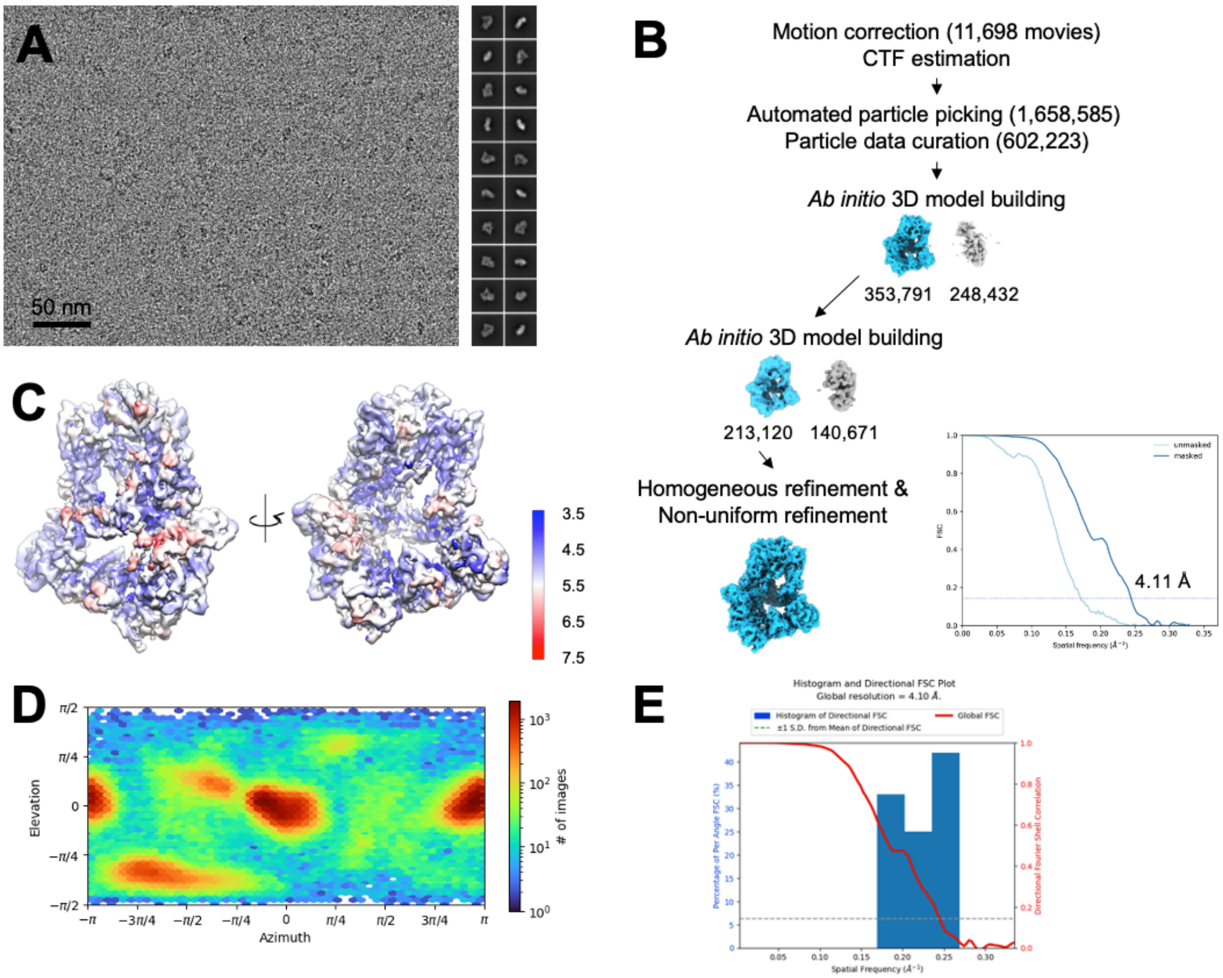

Figure S3. Cryo-EM image analysis of yeast apo Polζ. (A) Electron micrograph of the apo Polζ enzyme complex. Scale bar indicates 50 nm. Right boxes are representative class averages. Box side length is 347 Å. **(B)** Flowchart of the single-particle reconstruction. Resolutions were determined using the golden Fourier-shell correlation (FSC) at a cutoff of 0.143. **(C)** Local resolution estimation of the consensus cryo-EM reconstruction. Color map values are represented in Å. **(D)** Heat map of the distribution of projection directions or determined Euler angles of single-particle images. **(E)** Directional FSC plot of the 3D reconstruction.

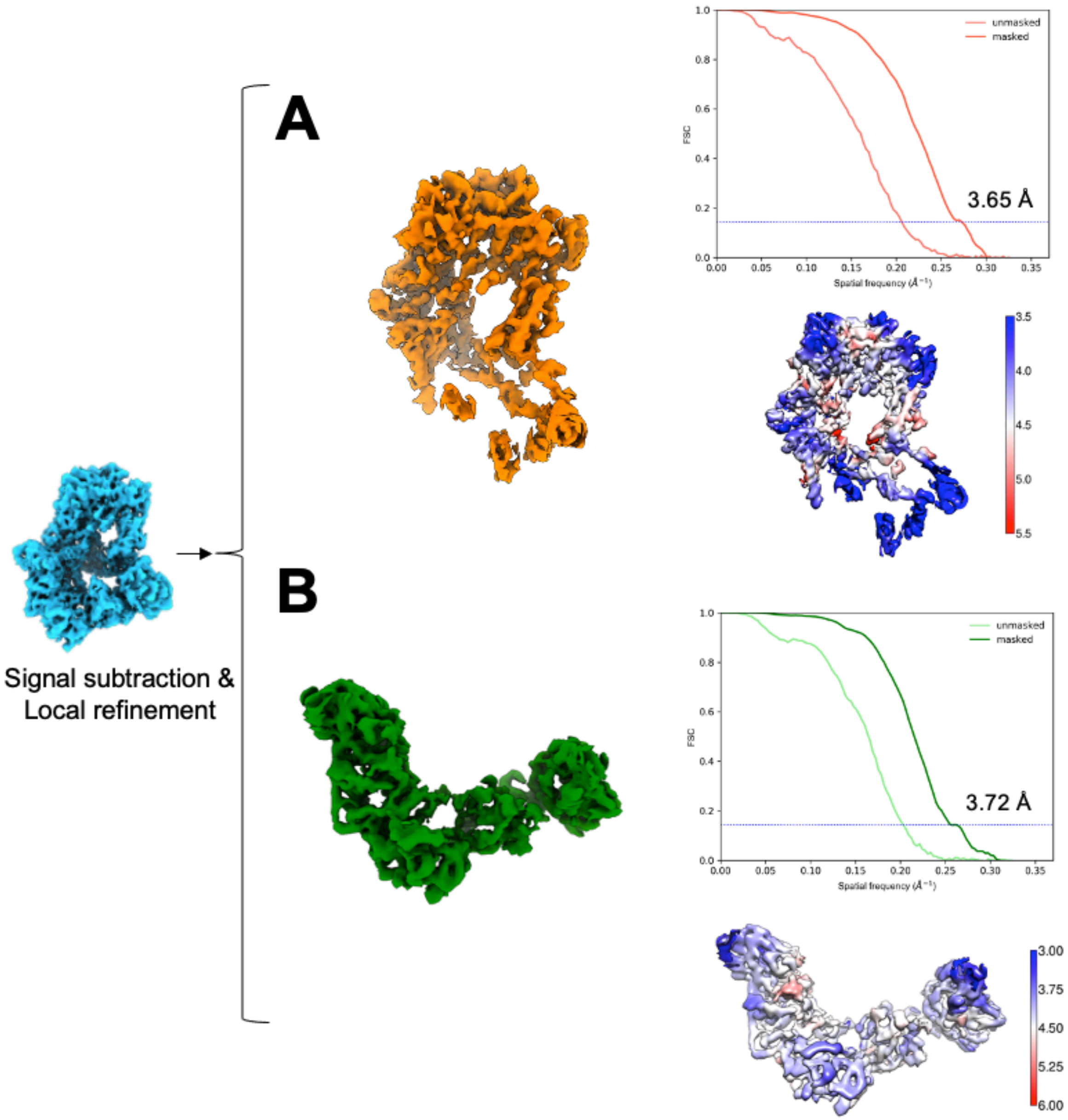

Figure S4. Signal subtraction and local refinement of the apo Polζ cryo-EM density. Locally refined cryo-EM densities of **(A)** Rev3 and **(B)** Rev7A-Rev7B-Pol31-Pol32. The Fourier-shell correlation (FSC) plots were determined using the golden FSC criteria at a cutoff of 0.143 (blue dash). The associated local resolution maps are shown with red-blue color ranges indicated along a bar in Å resolution.

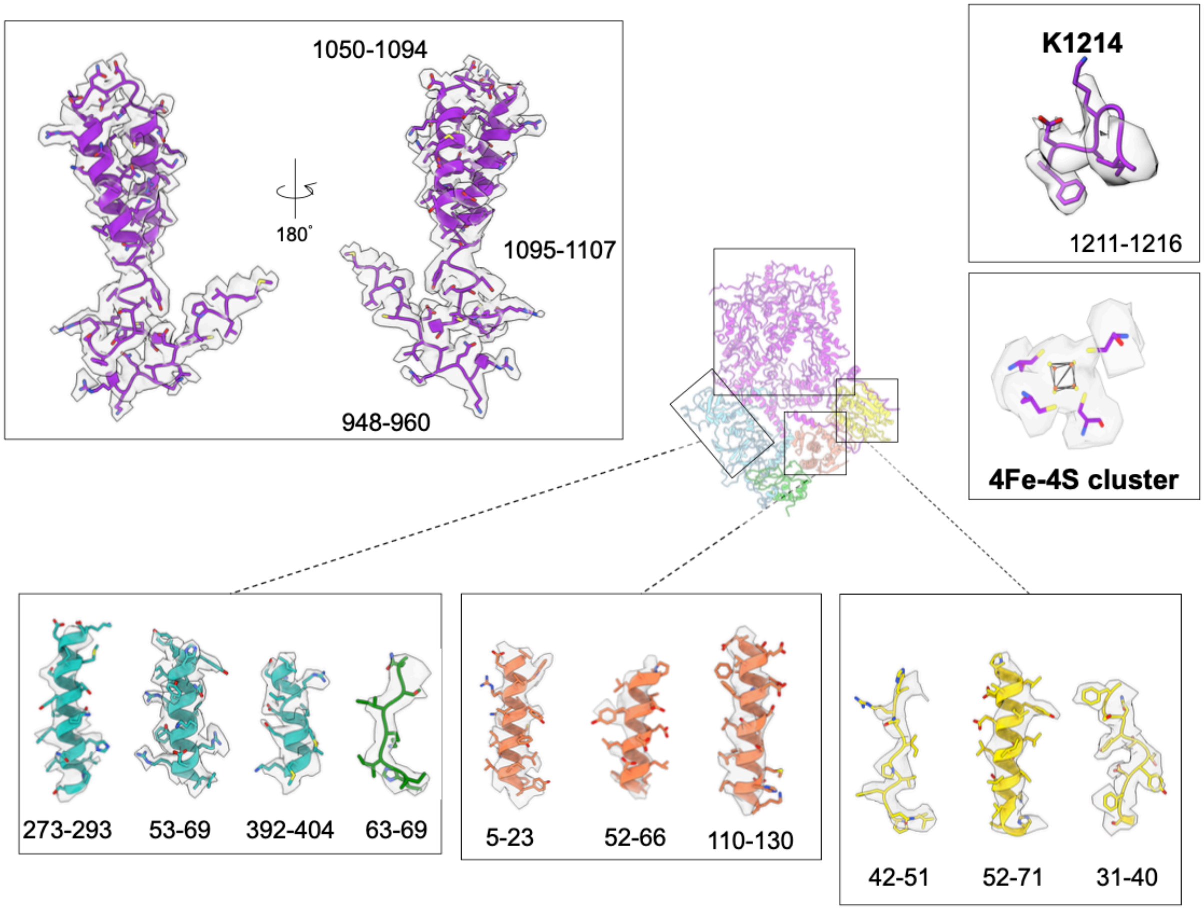

Figure S5. Cryo-EM density model fitting of yeast apo Polζ. Model building of the apo Polζ enzyme complex**.** Grey surfaces represent the cryo-EM densities. Colors are shown for Rev3 (purple), Rev7A (yellow), Rev7B (orange), Pol31 (cyan) and Pol32 (green).

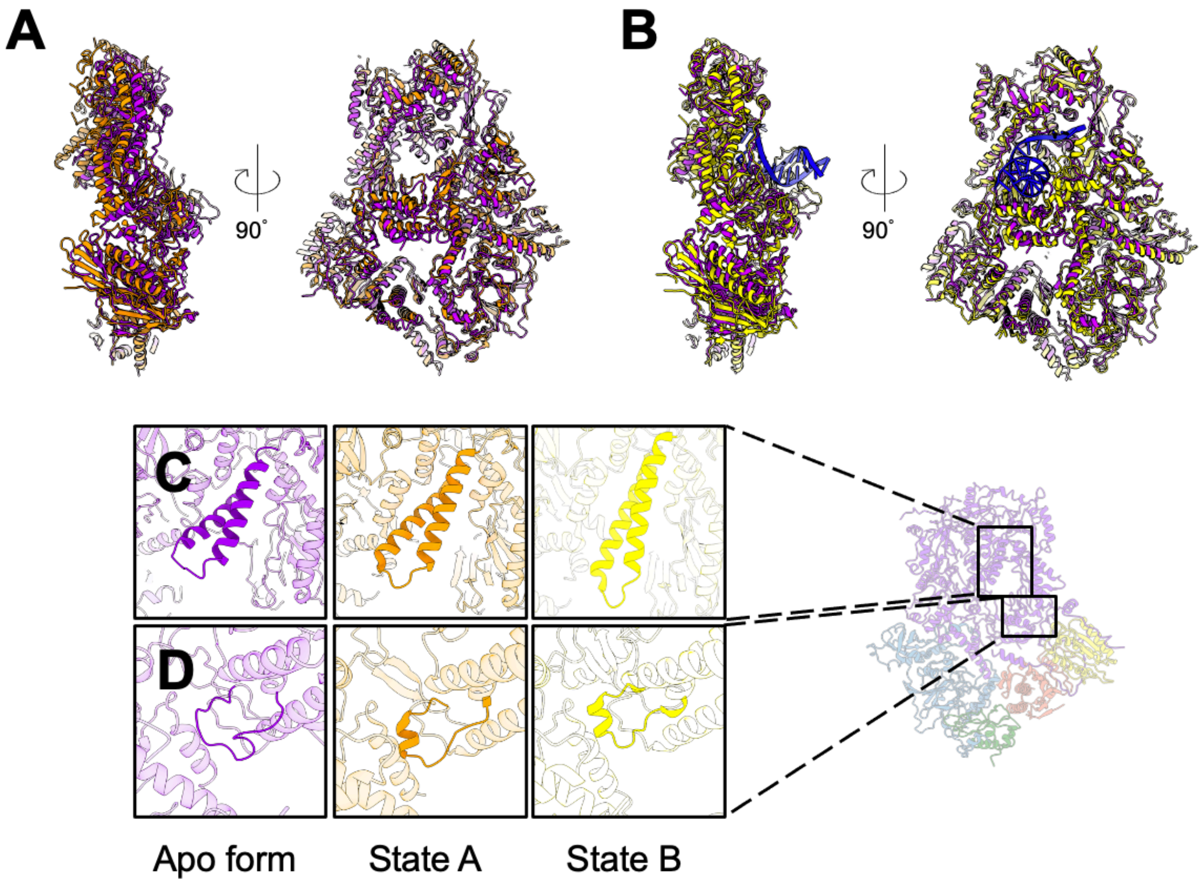

Figure S6. Structural changes associated with the transition from apo Polζ to DNA-bound Polζ. (A) Superposition of the structures of apo Polζ (purple) and Polζ in complex with a short DNA oligomer (state A; orange) (RMSD 1.34 Å). **(B)** Superposition of the structures of apo Polζ (purple) and Polζ in complex with a longer DNA oligomer (state B; yellow) (RMSD 1.36 Å). **(C)** States A and B correspond to the cryo-EM structures of Polζ bound to a short DNA oligomer (PDB code: 6V8P) and to a longer DNA oligomer (PDB code: 6V93). Apo Polζ and Polζ states A and B are shown in purple, orange and yellow, respectively. The movement of the helix-turn-helix motif (1050-1094 aa) is highlighted. **(D)** Same as B but with the conformational change of the Rev3 loop (1326-1344 aa) highlighted.

S. cerevisiae	YESPSQTLPIFDAKGIETVRRDGIPAQQKIIE	1232
H. sapiens	YETLDQKDPVFDAKGIETVRRDSCPAVSKILE	2871
B. bruxellensis	YEFEDQKEPIFDAKGIETVRRDGIPAQQKIME	1338
S. microadriaticum	WMSPSDVAPIFDAKGIETVRRDQCAATQHILR	1251
C. metapsilosis	YEYEDQVTPKFDAKGIETVRRDGIPAQQKITE	1413
N. crassa	YESRDQTVPVFDAKGIETVRRDGTPAEQRIEE	1506

Figure S7. Multiple sequence alignment of Rev3 across different species. The conserved lysine is highlighted in red.

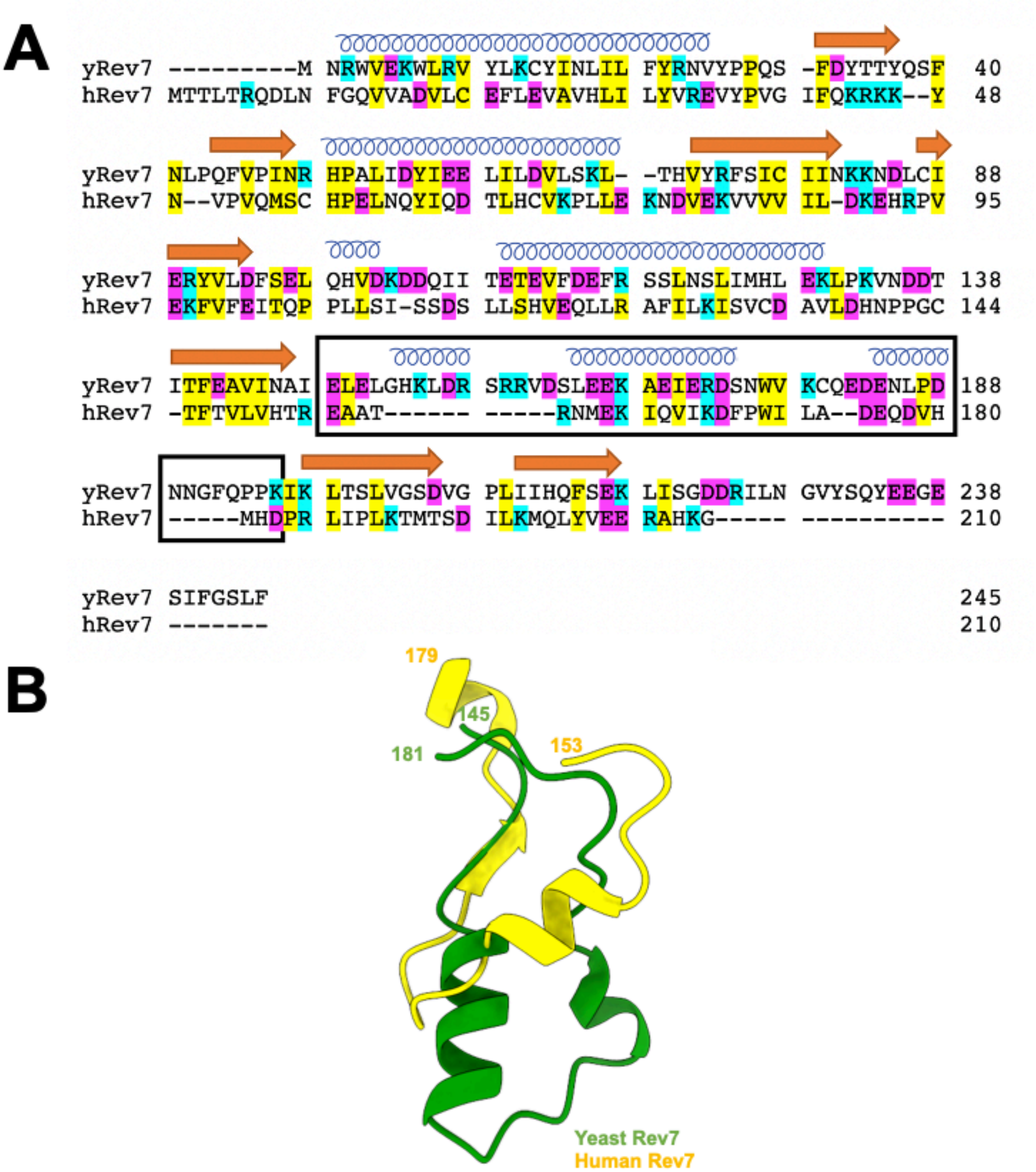

Figure S8. Amino acid sequence alignment and structures of *Saccharomyces cerevisiae* and human Rev7 proteins. (A) Aligned amino acid sequences of yeast and human Rev7. Springs and arrows represent helices and strands, respectively. Residues that are similar (yellow), positively charged (cyan) and negatively charged (purple) are highlighted. The safety belt region is enclosed in a black rectangle. **(B)** Structures of the safety belt regions of yeast (dark green) and human (yellow) Rev7.

Movie S1. Morph of the Polζ structures in different states. Morph structures include apo Polζ and Polζ bound to a short DNA oligomer (state A) and to a longer DNA oligomer (state B).
